# One Giant Leap for Categorizers: One Small Step for Categorization Theory

**DOI:** 10.1371/journal.pone.0137334

**Published:** 2015-09-02

**Authors:** J. David Smith, Shawn W. Ell

**Affiliations:** 1 Department of Psychology, Georgia State University, Atlanta, GA, United States of America; 2 Department of Psychology, University of Maine and Maine Graduate School of Biomedical Sciences & Engineering, Orono, ME, United States of America; University of Bath, UNITED KINGDOM

## Abstract

We explore humans’ rule-based category learning using analytic approaches that highlight their psychological transitions during learning. These approaches confirm that humans show qualitatively sudden psychological transitions during rule learning. These transitions contribute to the theoretical literature contrasting single vs. multiple category-learning systems, because they seem to reveal a distinctive learning process of explicit rule discovery. A complete psychology of categorization must describe this learning process, too. Yet extensive formal-modeling analyses confirm that a wide range of current (gradient-descent) models cannot reproduce these transitions, including influential rule-based models (e.g., COVIS) and exemplar models (e.g., ALCOVE). It is an important theoretical conclusion that existing models cannot explain humans’ rule-based category learning. The problem these models have is the incremental algorithm by which learning is simulated. Humans descend no gradient in rule-based tasks. Very different formal-modeling systems will be required to explain humans’ psychology in these tasks. An important next step will be to build a new generation of models that can do so.

## Introduction

Categorization is an essential cognitive adaptation and a focus of research and formal modeling [1–7]. An ongoing debate considers whether one, unitary category-learning system underlies humans’ diverse categorization performances or whether a second system uses explicit-conscious cognitive resources to discover category rules [8–14]. We argue that this debate is clouded by traditional approaches to analyzing performance in categorization tasks which can obscure psychologically meaningful transitions in category knowledge. This article illuminates this issue by focusing on humans’ psychological transitions when learning category rules and by demonstrating the failure of existing formal models to explain these transitions.

Our article proceeds as follows. First, we contrast analytic approaches to rule-based category learning, specifying approaches (e.g., backward leaning curves) that reveal humans’ psychological transitions during rule learning. Second, we document a crucial limitation of these approaches but also provide the means to surmount it. Third, we confirm that rule-learning humans do show dramatic, sudden learning transitions that could suggest they have a qualitatively separate rule-learning system that depends on explicit rule discovery. Yet this interpretation must be tempered by what existing models say on the matter. Fourth, we show that a popular exemplar-based model of categorization, ALCOVE [15], cannot predict humans’ learning transitions in rule tasks. It is an important theoretical conclusion that exemplar models like ALCOVE qualitatively cannot explain humans’ learning trajectory in rule-based category tasks (though they predict learning well in other situations). Fifth, we show that even formal models incorporating a rule system, COVIS [8], fail to explain this trajectory, most likely because COVIS (like ALCOVE) incorporates gradual, trial-by-trial changes in the state of the model. It is a striking fact, providing psychological insight, that human rule learning need not be gradual. It is another important theoretical conclusion that radically different formal-modeling systems will be required to accommodate humans’ true psychology in rule-based tasks.

## Analytic Approaches to Rule-Based Category Learning

### Forward learning curves

Forward learning curves are ubiquitous in research on discriminative learning (operant discriminations, Pavlovian associations, etc.). Researchers simply aggregate the performance of subjects on Block 1, Block 2, and so on, from the beginning of learning forward, and observe the group gradually acquiring a task solution, a skill, a behavioral reaction. Forward learning curves also accompany descriptions of category learning by humans [16], rhesus macaques–*Macaca mulatta* [16], and pigeons—*Columba livia* [14]. These examples are drawn from research by one of us—our discussion is deliberately self-critical.

It is well known, though, that forward learning curves have serious weaknesses as descriptions of learning. Estes [17] warned early on that mean curves could arise from an infinite variety of individual curves. He concluded that averaged data generally cannot address the form of individual learning functions. This theme is often echoed [18–20].

Given our focus on rule-based category learning and the psychological transitions therein, we state clearly the problem that forward learning curves pose in our area. Rule learning may occur suddenly by rule discovery, but participants may discover their rules at different times. By graphing average performance at each block, one likely displays the average of rule-using and rule-lacking participants, providing an empirical picture of what *no participant was doing*. By modeling averaged performance, one models something that has no basis in the psychology of any human mind.

To illustrate this problem, we produced a simulation in which hypothetical participants learned instantly through a process that in a human we would call rule discovery, but in which different hypothetical participants achieved their rule discovery at different blocks in the task. More specifically, the true, underlying probability of a correct categorization response (hereafter referred to as *competence*) by the hypothetical participants was manipulated so that it suddenly surged from chance to optimal accuracy across successive trial blocks.

In the simulation, 50 hypothetical participants completed 100 6-trial blocks of categorization performance. The block during which the surge in competence occurred was normally distributed (M = 50, SD = 32) thereby allowing for variability in the block during which hypothetical participants had their “insight”. Prior to the surge in accuracy, the competence of each hypothetical participant was set near chance correct-categorization performance (normally distributed, M = .5, SD = .08). The surge in accuracy was modeled by resetting competence to be near 1.0 (SD = .08, with a maximum level of 1.0). These variations in competence ensured that performance was not always precisely at chance or ceiling. Note that no actual task was simulated. Rather, each hypothetical participant was assumed to respond, on average, at an accuracy rate equal to their competence (e.g., a hypothetical participant with a competence of .54 would be correct on .54 of the trials, on average). Our intention was only to examine the forward learning curve that emerges when the competence of individual learners increases suddenly but at different blocks for different participants.

**Fig 1:**
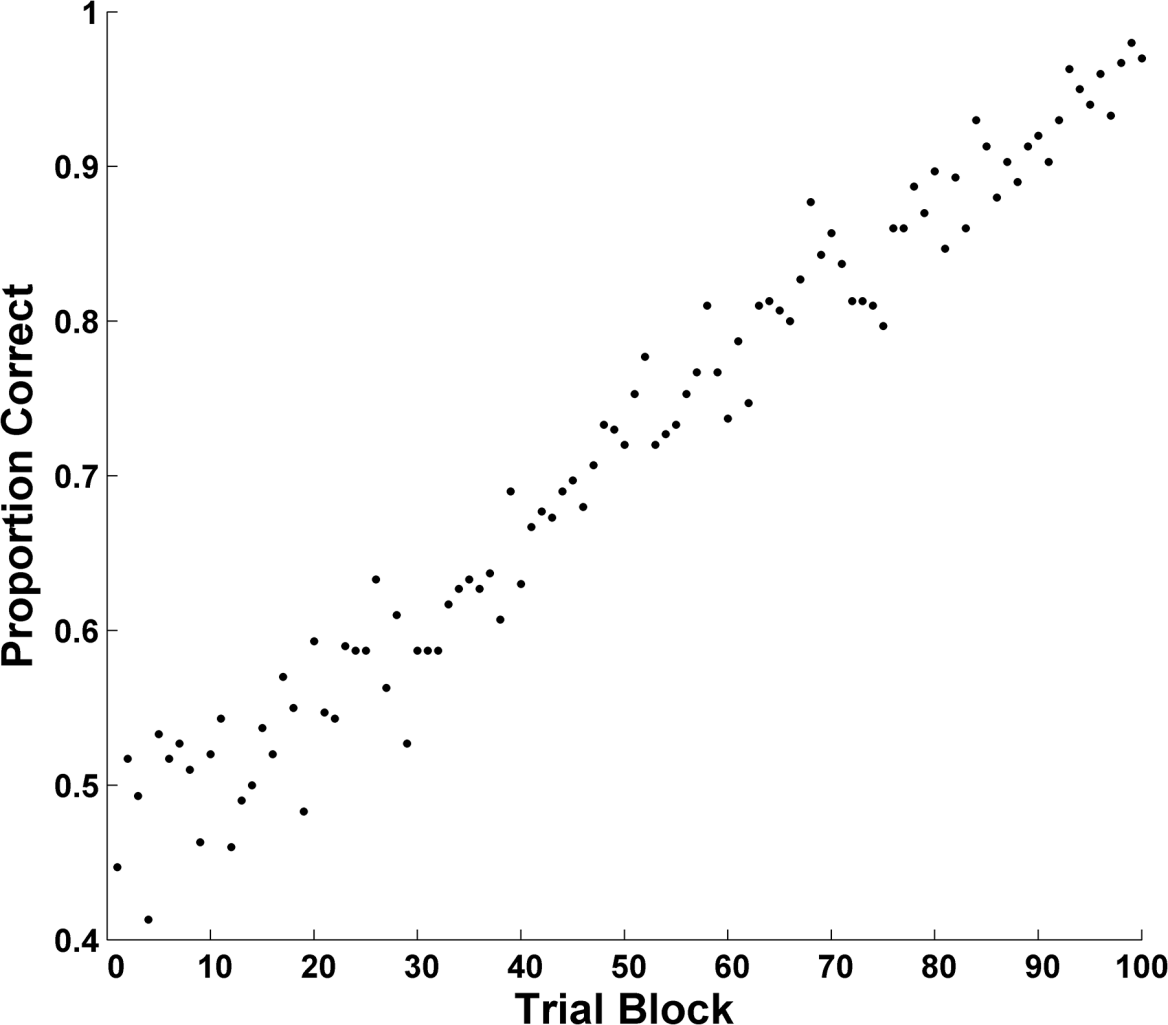
Forward Learning Curve: Sudden, Discontinuous Learning. The forward aggregate learning curve of 50 hypothetical participants with a discontinuous underlying learning process.

The results were averaged—block by block—across hypothetical participants and the resulting forward learning curve is shown in Fig 1. The learning curve misstates the course of learning and the underlying psychology of every hypothetical participant. And though one could model this aggregate curve with a model like ALCOVE, one would be modeling aggregated performance levels that bore no relation to the performance of any hypothetical participant. One sees that the forward-aggregate curve cannot be used to explore the psychology of rule-based category learning—*if humans show psychological transitions of the kind considered in this simulation*.

But there is a tension here. Fig 1‘s curve would seem to be very friendly to the gradual state changes that drive many formal models of categorization. Therefore, modelers may gravitate to forward learning curves and the gradual acquisitions they seem to reveal. They may do so even considering rule-based category tasks for which forward curves might be inappropriate. Resolving this tension is an important goal of the present article, for we will understand human categorization more clearly on resolving it.

### Backward learning curves

Hayes [21] also noted that forward learning curves are “quite irrelevant to basic problems of learning theory.” He said that only individual instances of learning by individual participants could address those problems. In perfect alignment with the theoretical problem of rule-based category learning in particular, he took on the challenge of showing abrupt rises in mastery that might occur at different times for different participants. His solution was the backward learning curve (BLC).

**Fig 2:**
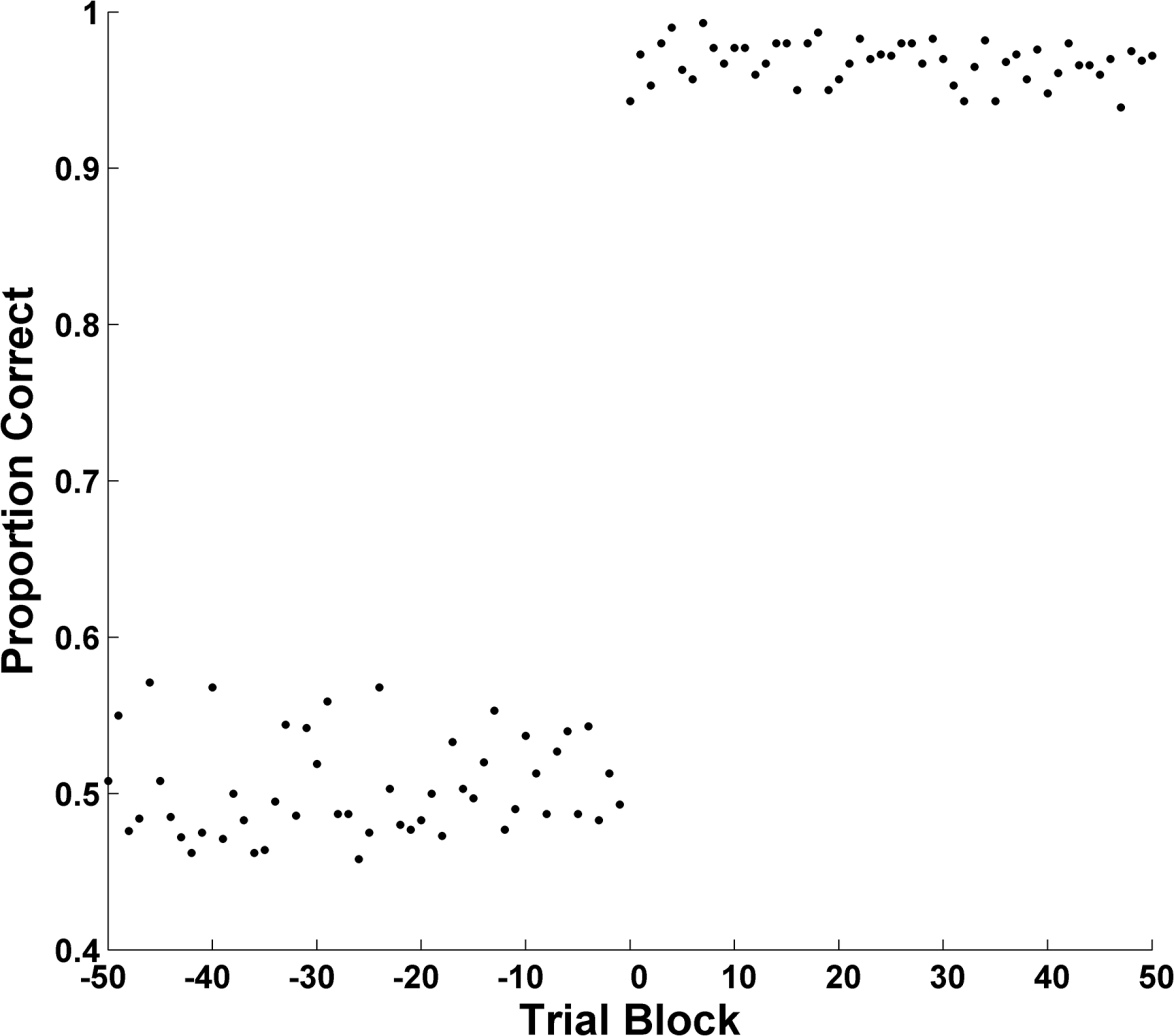
Backward Learning Curve: Sudden, Discontinuous Learning. The backward learning curve of 50 hypothetical participants with a discontinuous underlying learning process.

To construct a BLC, one defines a learning criterion and aligns the criterion block of all participants as Block 0. Then, one can look backward to Block -1 just before criterion, to Block -2, and so forth, discerning the possibly consensual path toward criterion. To illustrate the BLC, we reran the simulation just described. But now we aligned the data at the block during which each hypothetical participant began a long run with performance above criterion (Block 0). Criterion was defined as .83 correct. Then, after that alignment, we averaged performance across hypothetical participants. Clearly, Fig 2 captures the learning transition that actually dominated this simulation. This depiction of sudden learning is completely different from the depiction of apparently continuous learning shown in Fig 1. The BLC is far more illuminating in a case of this kind. Moreover, the BLC combines the power of a group analysis with the sensitivity of an individual-participant analysis, for it displays the psychological transition shown by every individual participant.

## Humans’ Performance in Rule-Based Category Tasks

It is an open question whether humans show qualitative performance shifts on rule discovery, and we address it now. We summarize data from two experiments among many that indicate these psychological transitions occur. Then we confirm that these transitions are robust to a serious limitation of BLCs, so that one can conclude that these transitions are psychologically real and theoretically crucial to acknowledge.

**Fig 3:**
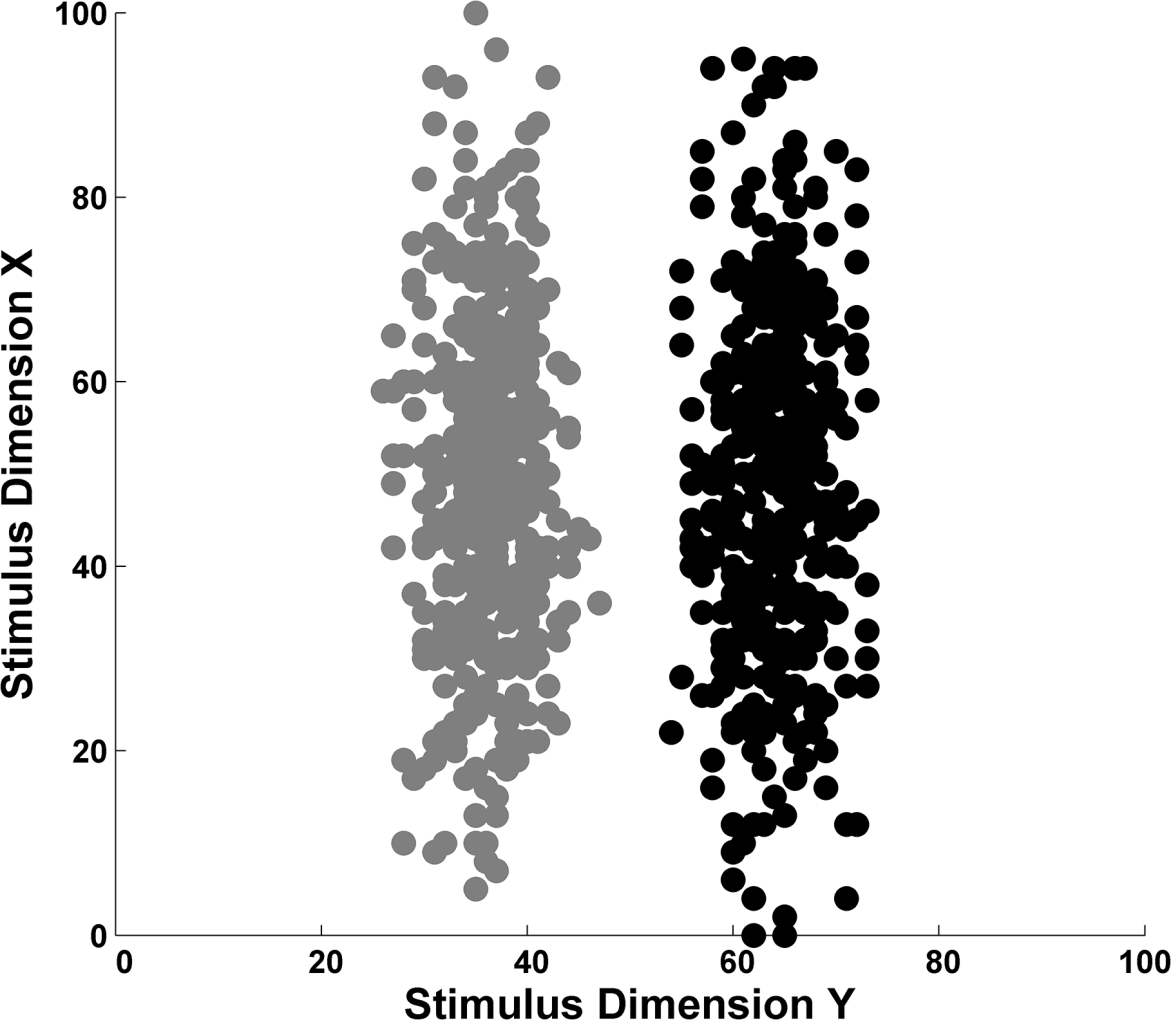
Scatterplot of a rule-based category learning task. The category A (gray) and B (black) exemplars were randomly sampled from separate bivariate normal distributions (for a detailed description of the stimuli, see 16).

Smith et al. [16] studied performance in Fig 3‘s rule-based task. Participants learned categories composed of sine-wave gratings varying in bar width and orientation. Humans strongly dimensionalized the stimuli and learned the task easily. An interesting feature of this study was that macaque monkeys showed a similar performance facilitation. This continuity across species could suggest that nonhuman primates share some structural components of humans’ capacity for explicit cognition.

**Fig 4:**
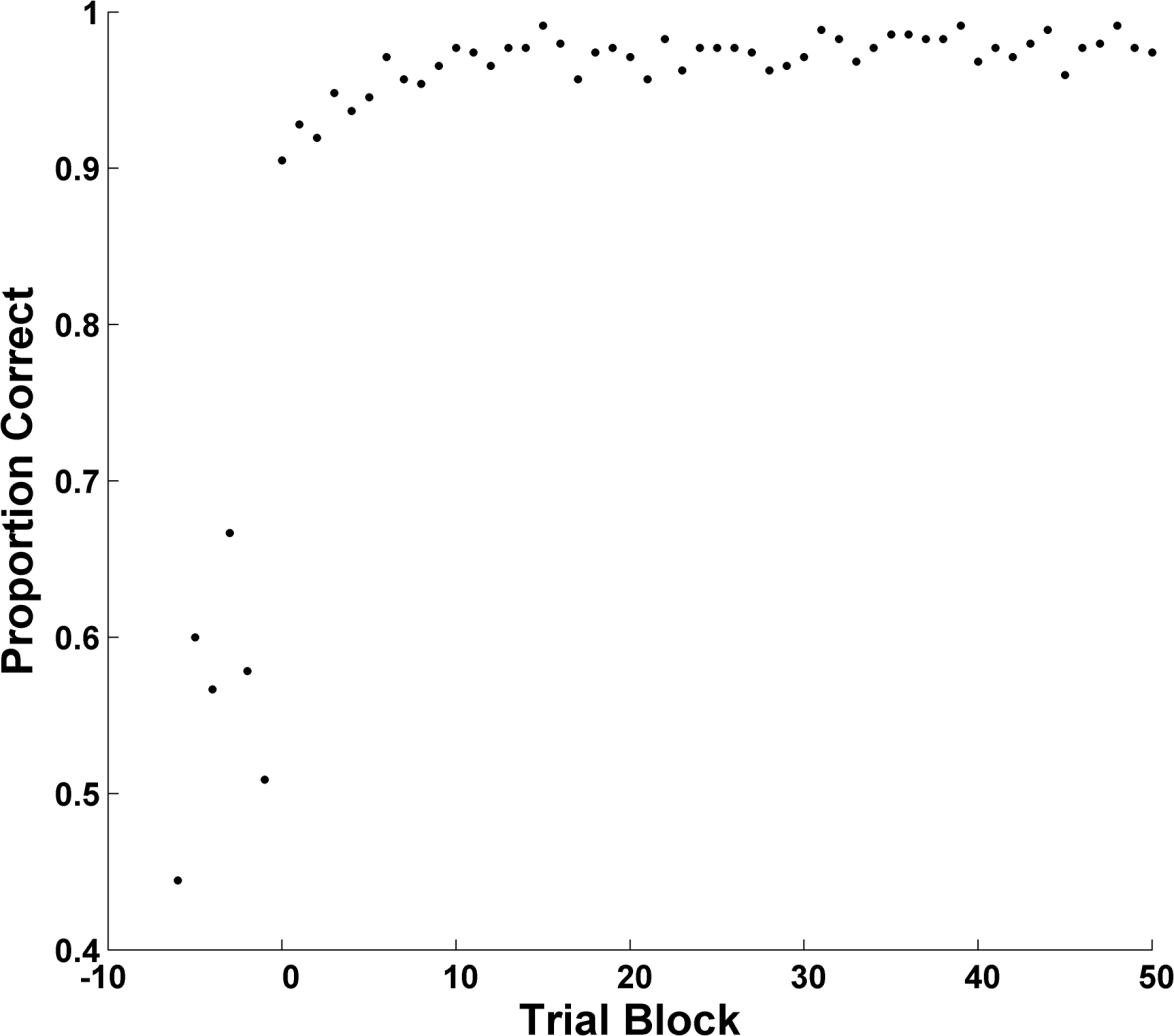
Backward Learning Curve: Smith et al. (2010). A backward learning curve summarizing the average accuracy of humans in [16].

Fig 4 summarizes performance in 6-trial blocks as a BLC. Humans clearly reached the criterion of .83 performance, and then sustained that performance level for many blocks thereafter. Moreover, they suddenly increased their categorization performance by .40 between two successive blocks in the rule-based task, from near chance to near ceiling. During their pre- and post-criterion performance, respectively, participants averaged .56 (*SD* = .070) and .97 correct (*SD* = .018)—a performance transformation of .41.

Smith et al. [22] studied rule learning in a similar rule task, but with categories composed of rectangles varying in size and in the density of lit pixels. An interesting feature of this study was that rule learning was easily possible when feedback was deferred until the end of each trial block and then given in summary form. This could suggest that humans can hold their category rule actively in working memory, so that the feedback can be interpreted equally well after each trial or after each trial block.

**Fig 5:**
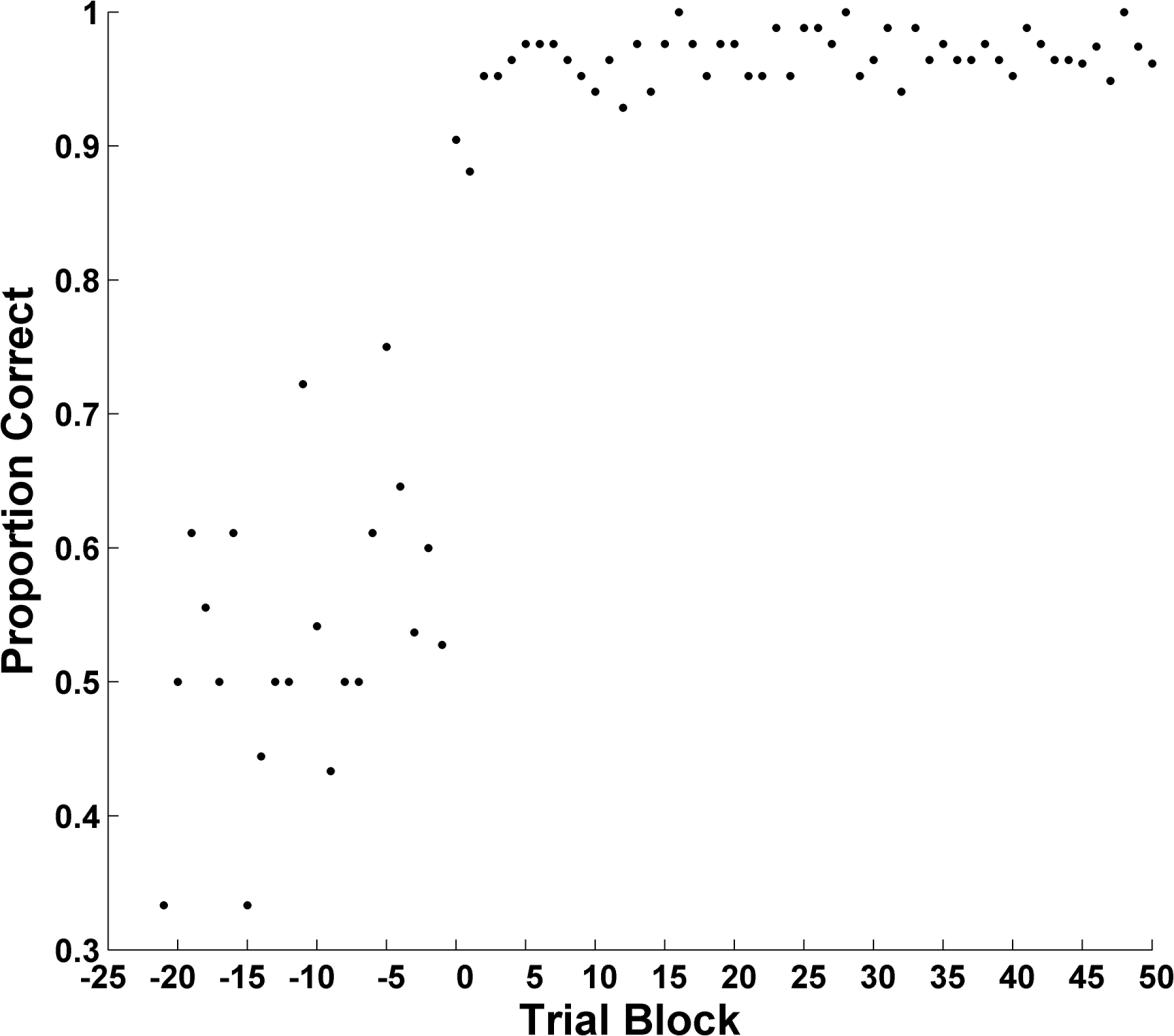
Backward Learning Curve: Smith et al. (2014). A backward learning curve summarizing the average accuracy of humans in [22].

Fig 5 summarizes performance in 6-trial blocks as a BLC. Again they reached the criterion of .83 performance and sustained it. They suddenly increased their categorization performance by .38 between two successive blocks in the rule-based task. During pre- and post-criterion performance, respectively, participants averaged .55 (*SD* = .095) and .96 correct (*SD* = .021)—the .41 performance transformation again.

## BLCs: Psychologically Real or Statistically Artifactual

However, these transitions require additional scrutiny because BLCs have limitations that are seldom noted. First, note that the blocks immediately preceding criterion cannot contain .83 or 1.0 performances. If they did, these blocks could become part of the criterion run that would be deemed to have begun earlier. Therefore, these blocks—sampled from a distribution of performance levels that is truncated high—could estimate performance artificially lower than participants’ true competence. Second, note that the blocks just post criterion cannot contain performances below .83. If they did, these blocks could become part of the pre-criterion phase that would be deemed to have lasted longer. Therefore, these blocks—sampled from a distribution of performance levels that is truncated low—could estimate performance artificially higher than participants’ true competence. Both limitations imply that the .41 transitions could exaggerate the change in underlying competence at criterion. As a matter of psychological interpretation, it is important not to do so. So we constructed a simulation to evaluate this exaggeration and estimate humans’ true psychological transition.

Similar to the earlier simulation (see Figs 1 and 2), we created hypothetical participants for whom we set the true proportion correct that would be achieved (i.e., competence). Pre- and post-criterion competence was independently set with 26 possible pre-criterion competence levels (.5 to .75 in steps of .01) and 26 possible post-criterion competence levels (.75 to 1 in steps of .01). Crossing all pre- and post-criterion competence values produced 676 hypothetical participants that varied in their transition in competence at criterion–i.e., their competence transitions ranged from 0 (.75-pre—.75-post) to .5 (.5-pre—1.0-post). As before, there was no actual categorization task. Instead, each participant was assumed to respond, on average, at an accuracy rate equal to their competence (e.g., a participant with a pre-criterion competence of .54 and a post-criterion competence of .9 would be correct on .54 of the trials pre-criterion and .9 of the trials post-criterion, resulting in a transition of .36, on average). In contrast to the earlier simulation, a participant’s competence was not randomly sampled from a normal distribution.

We simulated the performance of each hypothetical participant (i.e., each of 676 competence transitions) on 100 6-trial blocks. This simulation was repeated 10,000 times for each hypothetical participant with the criterion block varying across repetitions (approximately normally distributed, M = 50, SD = 16). We forced the criterion block to lie between blocks 25 and 75, though, to always leave 20 blocks pre- and post-criterion available for summarizing performance. The data from each repetition in which criterion was met (i.e., reaching an observed proportion correct of at least .83 that was sustained for 20 blocks) was averaged (across repetitions for a given participant) resulting in an estimate of pre- and post-criterion performance for each of the 676 underlying competence transitions.

This simulation would help to support a crucial inference. Now we knew the observed levels of pre- and post-criterion performances that result from many true levels of pre- and post-criterion competence. And we could read from the simulation’s results the underlying levels of competence that can produce the observed .55 and .96 levels of pre- and post-criterion performance that humans actually show in rule-based tasks.

**Fig 6:**
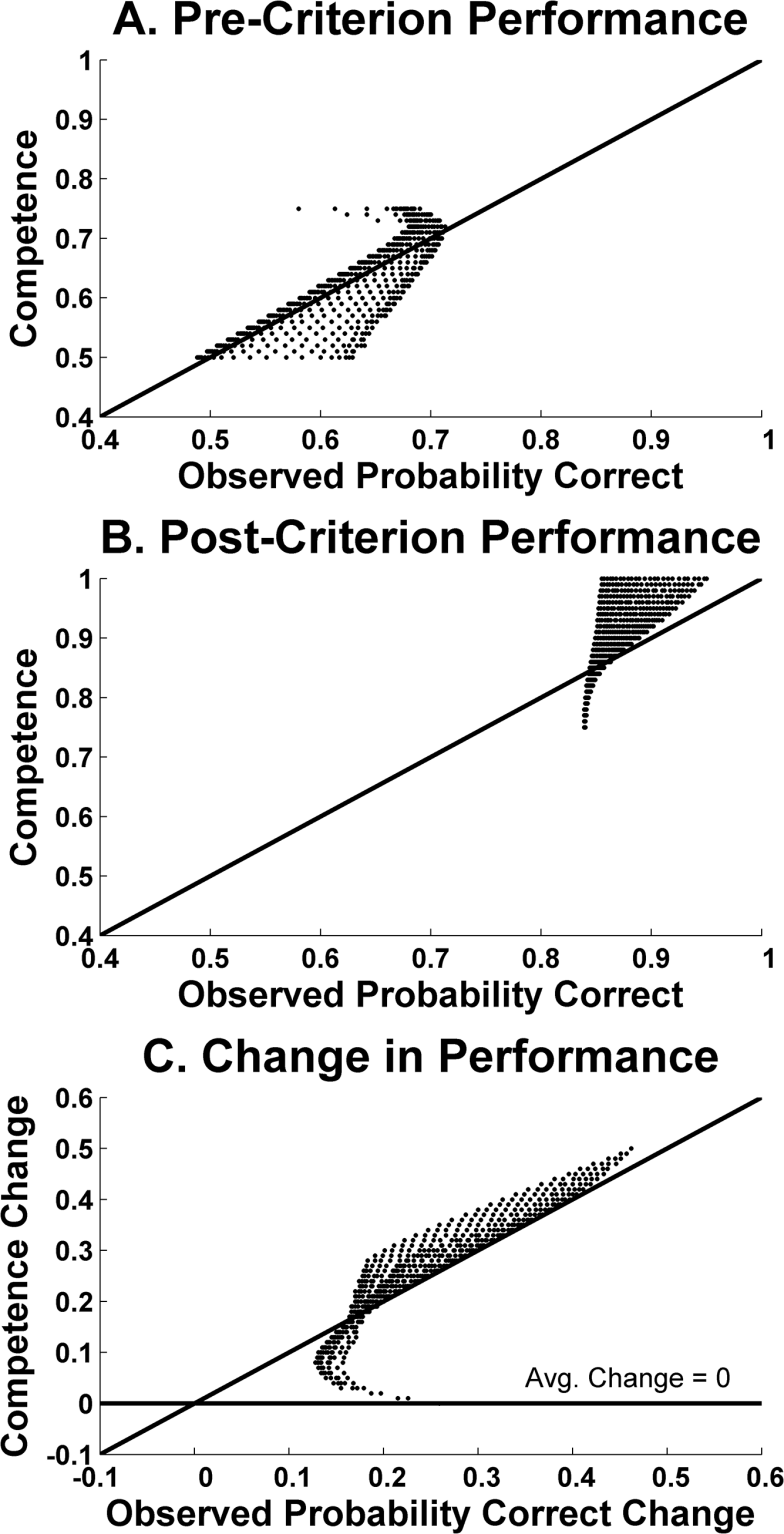
Results of simulations that varied independently true underlying competence during the pre-criterion phase and post-criterion phase of a hypothetical category-learning task. *A*. Underlying pre-criterion competence (Y-axis) plotted as a function of the pre-criterion performance actually expressed within the simulation (X-axis). *B*. Underlying post-criterion competence (Y-axis) plotted as a function of the post-criterion performance actually expressed within the simulation (X-axis). *C*. The change in underlying performance competence (Y-axis) plotted as a function of the pre-post performance transition actually expressed within the simulation (X-axis). In all panels, the diagonal line represents perfect correspondence between the performance produced by the simulation and its underlying-competence inputs. Each data point represents the average data from one of 676 simulated competency transitions.

In Fig 6A, the X-axis shows the pre-criterion proportion correct for the 676 hypothetical participants as they performed in the simulation. The Y-axis shows the pre-criterion competence that we had set for each participant. Participants that perform, like humans, at about .55 (X-axis), are drawn from a restricted population with competence at about .56 (Y-axis). There is almost no statistical artifact. The .55 is essentially a true score. The explanation for this is that the pre-criterion competence is so low that performance would rarely reach .83 or 1.0 in any case, and so any truncation effect in the distribution of performances is negligible.

Fig 6B shows the analogous result for post-criterion performance. Hypothetical participants that perform, like humans, at about .96 post-criterion (X-axis), are drawn from a restricted population with competence near .96 (Y-axis). There is no mismeasurement in the .96 estimate. It is a true score again.

Fig 6C shows the simulation’s combined result using performance transitions from pre- to post-criterion. (X-axis: performance transitions by the hypothetical participants as they performed in the simulation; Y-axis, their competence transition).

Participants that improve by about .40 from pre- to post-criterion (X axis), like humans do, are drawn from a restricted population with a competence change of .40 or even higher (Y-axis). The observed performance change reflects perfectly the underlying competence change.

We conclude that essentially all of humans’ .41 performance transition is due to a sudden shift in category knowledge and its application. Humans transform their performance at criterion over just 6 trials. Researchers in this area must realize that the use of forward learning curves is not sufficient or appropriate in the area of rule-based category learning. If one analyzes rule tasks in this way, or models data in this way, one is being profoundly deceived by misleading gradualism in learning. A sufficient explanation of humans’ rule learning will have to explain the leaps of categorization competence. A sufficient model of categorization will have to reproduce them. The search for the sufficient explanation and model raises important theoretical questions for the categorization literature, as we will see next.

### BLCs and ALCOVE

These insights let one approach the multiple systems-single system issue in a new way, by evaluating the sufficiency of single-system models in this area. Exemplar theory has been a dominant influence in categorization for decades. Kruschke’s [15] ALCOVE model has become the dominant representative of exemplar models. Thus, it is of interest to know whether exemplar theory, and its ALCOVE model, can reproduce the transitions that humans show at criterion in rule-based tasks. That is, it is important to establish the sufficiency of exemplar theory and ALCOVE regarding rule-based categorization data.

ALCOVE assumes that category decisions are made by computing the similarity of the stimulus to memory representations of all previously seen exemplars. ALCOVE has four parameters. The sensitivity parameter (c) is a measure of overall discriminability. The determinism parameter (φ) specifies the consistency of responding. Two learning rates (λ_w_ and λ_a_) determine how quickly the exemplar-category associations and attention weights are learned. For the present simulations, c varied from .5 to 6.5 with increments of 1.0, φ varied from .5 to 4.5 with increments of 1.0, λ_w_ varied from 0 to .15 in steps of .01, and λ_a_ varied from 0 to .15 in steps of .01 for a total of 8,960 parameter combinations (7 X 5 X 16 X 16). These parameter ranges were drawn from published literature and are representative of parameter values that have been used to simulate human behavioral data [15, 23, 24]. All simulations were performed using Matlab and the code is available as supporting information.

ALCOVE was simulated for 100 6-trial blocks on the Fig 3 task to find its blocks-to-criterion, its pre- and post-criterion performance levels, and its performance transition at criterion. Criterion was defined as the block with proportion correct > = .83 when that performance level was then sustained for the remainder of the 100-block simulation run.

No direct restrictions were placed on pre-criterion accuracy. However, in studying ALCOVE’s BLCs, statistical artifacts must still be considered. The definition of criterion will truncate high and artificially lower pre-criterion accuracy, and truncate low and artificially raise post-criterion accuracy. As noted previously, this is a significant potential limitation of the use of BLCs, one that must be addressed with care for proper interpretation. In the previous simulation (Fig 6), we could easily look behind these statistical artifacts and subtract away their effects. We were omniscient because we knew the competence of the simulated categorizers because we created them. But that is not true here. Therefore, ALCOVE’s transitions will partially be artifactual and we must find another way to separate these artifacts away. An additional contribution of the present article is that it specifies techniques that can let researchers manage and surmount this potential limitation of BLCs.

Here we did this as follows. ALCOVE assumes that the weights between network layers are updated by a process of gradient descent on error. But as the pre- and post-criterion blocks are completed by the model, the state of the model’s network (i.e., the numerical values of the attention and association weights) is known. This network information lets us find the model’s underlying competence in that state. More specifically, for each parameter combination, we saved the network state at the end of the pre-criterion block and the network state at the end of the post-criterion block. Next ALCOVE was simulated for an extended time (100 blocks), with the learning mechanisms disabled, using the saved pre- or post-criterion weights. The act of ‘freezing’ ALCOVE’s weights at the end of the pre- and post-criterion blocks enabled us to estimate ALCOVE’s long-run competence given the pre- or post-criterion state of the model. Chance events, statistical artifacts, and criterion definitions could not bias these performance estimates. These runs gave us the equivalent of the 20-block look back and look forward that let us measure humans’ true psychological transition in the simulation of Fig 6.

We averaged our results over 500 repetitions, separately for each of the 8,960 parameter configurations. In this averaging, we excluded repetitions in which criterion was met during the first block, never met, or was met only in the run’s last 25 blocks. The two exclusions are obviously necessary. The third exclusion ensured that ALCOVE was challenged to sustain performance over many blocks at or above the criterion level, just as we required of humans in Figs 4 and 5. To be clear, we did not average across the different parameter configurations, but only across the replications of each single parameter configuration.

A reviewer suggested we acknowledge that our procedures are somewhat more complex than when one estimates model parameters from data. In this analysis, we are not fitting data, but studying the behavior of models at important transition points. We are evaluating carefully the extent of statistical artifacts at those transitions. We are also introducing innovative approaches to understanding the underlying performance competence of a model as it technically meets criterion or undergoes a transition. These are new and constructive approaches toward studying category models, and they warrant some complexity.

**Fig 7:**
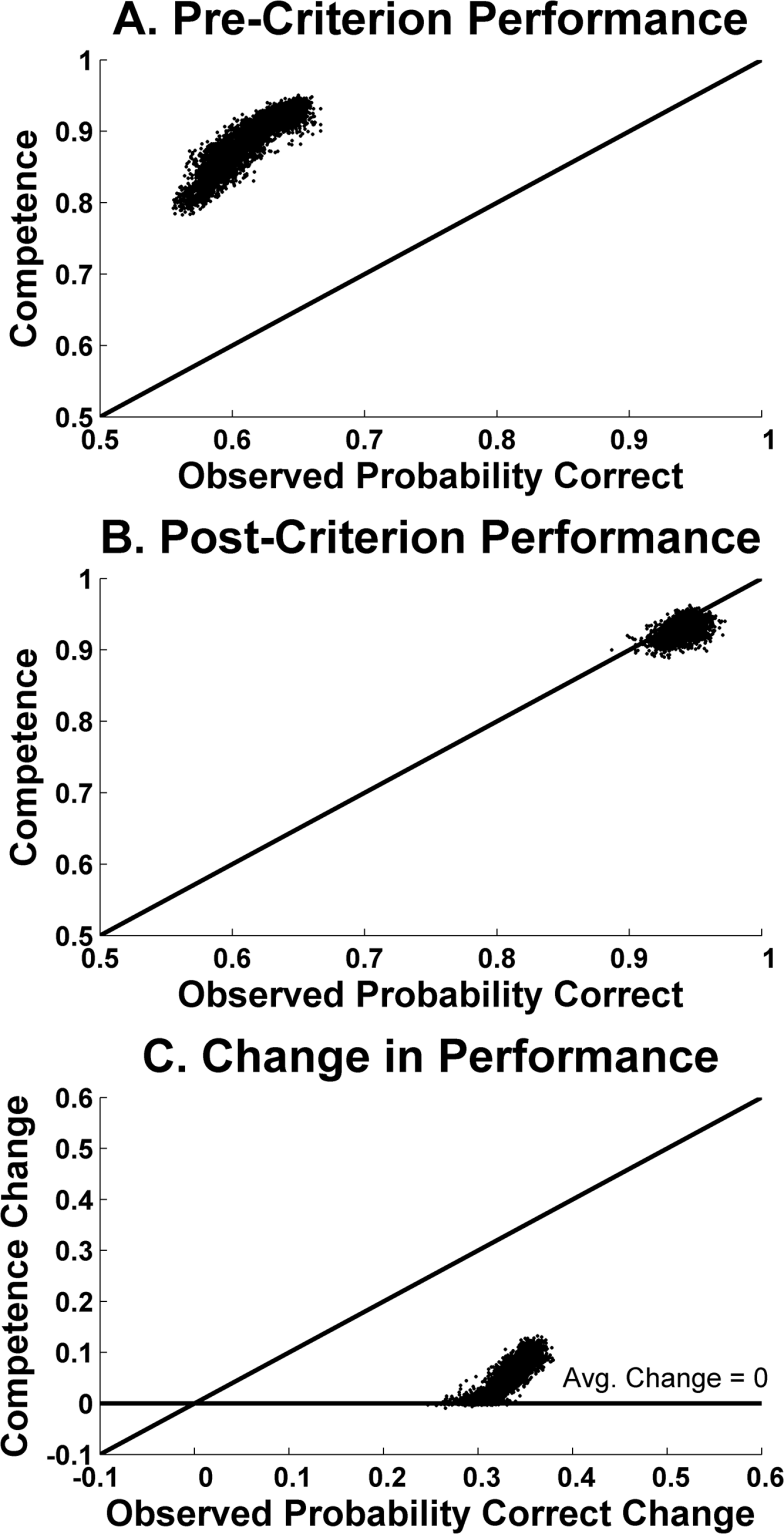
Results from the ALCOVE simulations, with ALCOVE learning the Fig 3 RB categorization task from a variety of parameter configurations. *A*. Underlying pre-criterion competence (Y-axis) plotted as a function of the pre-criterion performance actually expressed within the simulation (X-axis). *B*. Underlying post-criterion competence (Y-axis) plotted as a function of the pre-criterion performance actually expressed within the simulation (X-axis). *C*. The change in underlying performance competence (Y-axis) plotted as a function of the change in performance actually expressed within the simulation (X-axis). In all panels, the diagonal line represents perfect correspondence between the observed performance produced by the simulation and its underlying true competence at the point of producing that observed performance. Each data point represents the average data from one of 8,960 simulated competency transitions. All simulations were performed using Matlab and the code is available as supporting information.

Fig 7 summarizes the results of ALCOVE (in an identical format to Fig 6). Fig 7A (X-axis) suggests that ALCOVE predicts low pre-criterion performance (about .6), consistent with the truncation problem by which .83 and 1.0 proportion corrects cannot lie in that block of the BLC. Unless treated carefully, this would give the false appearance that ALCOVE successfully predicts a large pre- to post-criterion performance transition. In fact, on freezing ALCOVE’s performance network at the end of the pre-criterion block, we estimated with no truncation bias the model’s competence at that point in learning. Fig 7A (Y-axis) shows that in reality ALCOVE has high competence at that block, confirming a strong statistical artifact one block before criterion, and confirming that ALCOVE in reality can only manage a very small transition at criterion.

Fig 7B shows that our estimates of ALCOVE’s performance agree whether taken at just the post-criterion block (X-axis) or in the long run by freezing ALCOVE’s network state at the end of the post-criterion block (Y-axis). This agreement reflects that performance must be sustained at a high level for the run to count as criterion, and so therefore we already know that the model will probably sustain criterial performance because it demonstrably did so to meet the definition of criterion in the simulation.

Fig 7C shows the true transitions that ALCOVE allows from pre- to post-criterion. The apparent change in performance pre- to post-criterion (Fig 7C, X axis) is large, so one might think the model produced a sharp transition. It is not so—those transitions are artifactual. The true transitions are very small (Fig 7C, Y axis) and they miss completely the profound psychological transitions humans show at criterion.

Given the importance of the exemplar perspective within categorization science, and of ALCOVE as the dominant exemplar model, we undertook a complementary analysis. Our goal was to provide a converging viewpoint on ALCOVE’s gradualism and its failure to accommodate humans’ sudden leaps in categorization performance.

In this analysis, we did fit ALCOVE to the data already shown in Fig 5. This approach accommodated a reviewer’s very constructive suggestion that we examine ALCOVE’s behavior at the very parameter settings that let it fit observed behavior. Accordingly, we found ALCOVE’s BLC back and forth from the arrival at criterion when it was using those best-fititng parameters (c = 2.75, φ = 1.75, λ_w_ = .13, λ_a_ = .86). To do so, we found the block (out of 100) during which criterion accuracy was achieved and maintained for the remaining blocks. The results were averaged over 1,000 replications at those parameter settings. Similar to the Fig 7 simuations, replications in which criterion was met prior to the 5^th^ block, was never met, or was met only in the run’s last 25 blocks were excluded.

**Fig 8:**
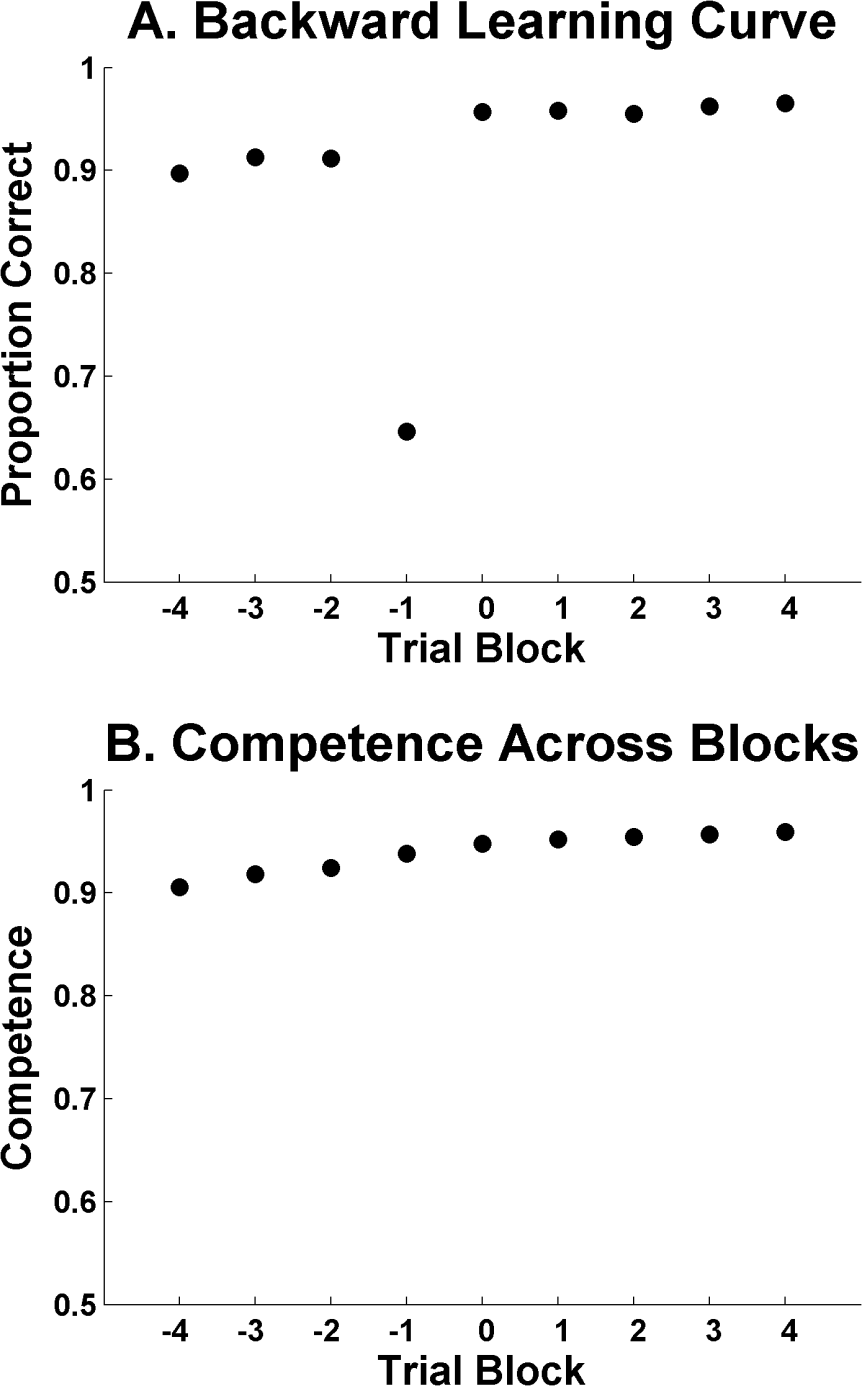
Results from the ALCOVE simulations using parameters estimated from the Fig 5 data. *A*. Backward learning curve summarizing ALCOVE’s average accuracy. *B*. Underlying competence at each block.

Fig 8A shows the result. ALCOVE badly misses several aspects of what humans do (Fig 5). ALCOVE’s pre-criterion performance is far too high. Its dip below .83 at Block -1 is obviously an artifact caused by the fact that the pre-criterion block is definitionally not allowed to be as high as .83. So the dip—and, more importantly, the leap to criterion—is purely the technical outcome of the formal definition of criterion. It appears as though ALCOVE did not experience any leap in true competence at criterion, as humans clearly do. By interpolation, one might judge that ALCOVE’s true competence at Block -1 is above .90, implying that full gradient descent gradualism was in force here, with ALCOVE gradually rising toward its asymptotic performance. With these best-fitting parameter settings, ALCOVE may have still been behaving according to its basic operating axiom of gradualism, even in this example that seemingly showed a substantial, but artifactual, performance leap.

To confirm this, we also froze ALCOVE’s weights at the end of each of the blocks in Fig 8A and ran the model for 100 consecutive blocks, in order to estimate ALCOVE’s true long-run competence at the model’s state for each block, with all technical definitions of pre- and post-criterion set aside.

Fig 8B shows the result. ALCOVE, in sharp contrast to humans, shows no leap in competence at criterion. ALCOVE’s pre-criterion competence was above .90. There was no insight, no realization, no hypothesis confirmed, no discovery made. Of course there wasn’t. This isn’t the way ALCOVE does or can work. Instead, ALCOVE is showing gradient-descent gradualism as its competence increases toward criterion. Its leap in Fig 8A is essentially all artifact. We found that exactly the same result obtained even when we redid this analysis but somewhat loosened the demands on ALCOVE to show an extended criterion run (i.e., requiring ALCOVE to maintain criterion accuracy for 20 blocks). This analysis is highly conservative, because it represents an analysis of an apparently large criterion leap by ALCOVE. This analysis also served the constructive function of illustrating ALCOVE’s behavior using the very parameters it uses in fitting humans’ performance data. In all respects, this analysis confirms and amplifies the general conclusions of the article.

ALCOVE’s gradualism is not an artifact of the parameter ranges tested in these simulations. The simulations covered generously the parameter configurations that have been used to fit human performance. That is, they covered the configurations that are relevant to the study of human categorization. In our view, this approach gave ALCOVE its best chance of succeeding in showing humans’ leaps in performance. It could not do so, even when we focused on the very parameter settings that ALCOVE uses to fit human data.

Nor does ALCOVE’s gradualism arise because we cast our simulation net too broadly, and were including “weird” configurations not relevant to the study of human category learning. It would have been poor practice to take this approach, or to sample ALCOVE’s parameter ranges fully exhaustively, because this would have justified the weirdness criticism. Instead, we deliberately chose the range of parameter settings most relevant to the study of human category learning. In any case, the conclusion from the simulations is not affected by the “weirdness” possibility. For ALCOVE fails in just the same way across the whole range of the simulation.

Nor do we believe that our grid-search scans missed some key parameter configurations that would have dramatically given ALCOVE human leaps in acquisition. Of course there is a vanishingly small possibility of this. However, in our combined 30 years of formal-modeling experience, we have never observed the performance surfaces running through parameter spaces to misbehave in this way. Worse, this criticism is limitless. One’s defense of ALCOVE could rise to the demand of a grid search to 3 decimal places, or 4 decimal places, or more.

Nonetheless, we acknowledge that our enterprise required choices, and that different choices are possible. Illustrating this point, an excellent reviewer aimed a perfectly contradictory criticism at our ALCOVE simulations. He or she said that we had sampled too broadly, including many weird parameter configurations that might have put ALCOVE in a bad light, while also we had sampled ALCOVE too narrowly, so that we missed the excellent parameter configurations that might have existed in the nonhuman ranges of the parameters. Clearly, this is not an issue we can win, unless perhaps one says that we got the simulations about right because we were at risk both ways. But of course the criticism cannot have it both ways at once, either. Regardless, we acknowledge the reviewer’s wise sense of the difficulty of the choices required here.

Many readers, who observe dispassionately theoretical developments within categorization, will see that all of the foregoing criticisms are submerged in the grounding mechanism of the ALCOVE model. It is very clear that ALCOVE’s gradualism arises because it adjusts its weights quantitatively and gradually using gradient descent. One could try to remedy this gradualness using highly extreme parameter values (e.g., high learning rates) that might result in sudden large changes in accuracy. But these values could destabilize performance, disallowing the maintenance of criterion levels of performance [25, 26]. They might also engender criterion performance instantly, so that one could not observe for a different reason the performance transitions that humans show. These are illustrations of why patently nonhuman parameter configurations would not have been good choices in this study. Moreover, if ALCOVE were successful in creating performance leaps, but only using extreme parameter settings that are not remotely “human,” this would be an equally serious problem for that modeling approach. Really, the explanation for ALCOVE’s failure here is intuitive and should not be overthought: ALCOVE “learns” quantitatively and gradually, but humans in rule-based tasks learn qualitatively and suddenly.

### BLCs and COVIS

The character of the exemplar model’s failure indicated that the problem with current formal models might extend more broadly than just to exemplar models. So we also challenged the modeling framework COVIS [a prominent multiple-systems model with a rule-learning component—8] to reproduce humans’ performance leaps at criterion. We show now that this model also clearly fails to do so.

Ashby and his colleagues designed COVIS to incorporate an implicit, associative-learning process and an explicit, hypothesis-driven rule process in competition within a single modeling framework. Previous data and applications of COVIS suggest that the explicit process dominates in rule-based tasks [27, 28] unless it fails [29, 30]. Accordingly, we analyzed the behavior of COVIS’s explicit (rule) system only.

The COVIS explicit system assumes that category decisions are made by comparing the stimulus to a decision criterion that partitions the space into response regions (e.g., category A and B regions).The explicit system has four parameters that govern rule learning. The switching parameter (γ) determines the likelihood of switching attention away from the current stimulus dimension, the selection parameter (λ) determines the probability of selecting a new stimulus dimension, and the salience of a stimulus dimension is adjusted following correct (Δ_C_) and incorrect trials (Δ_E_). Both stimulus dimensions were initialized with a salience of .5. For the present simulations, γ varied from .6 to 12 (8 equally spaced values), λ varied from .05 to 10 (8 equally spaced values), and Δ_C_ and Δ_E_ were initialized to .01, .055, or .1. To facilitate the simulation, the Fig 3 stimuli were scaled along both dimensions by a factor of 47.75 (resulting in a stimulus range of 0 to 1 on X and 0 to 2.57 on Y). The initial placement of the decision criterion on each dimension was set to a random starting value within the range of stimuli and was learned by a modified gradient descent algorithm [i.e., the delta rule with a momentum term and learning rate annealing—8]. This algorithm has three parameters that govern criterion learning: the initial learning rate, momentum, and annealing rate). For the present simulations, the initial learning rate varied from .06 to .5 (10 equally spaced values), momentum was set to .732 or .932, and the annealing rate was set to 42 or 56. All possible parameter values were crossed resulting in 23,040 parameter combinations (8 X 8 X 3 X 3 X 10 X 2 X 2). These parameter ranges were drawn from published literature and are representative of parameter values that have been used to simulate human behavioral data [15, 23, 24] All simulations were performed using Matlab and the code is available as supporting information.

COVIS assumes that the salience of each stimulus dimension and the decision criteria on each dimension are adjusted during the course of learning. Thus, the numerical values of these salience and criteria define the network state of the model and are known at the end of the pre- and post-criterion blocks. As with ALCOVE, this network information can be used to find the underlying competence of COVIS in that state. Just as we did with ALCOVE, we saved the network state of COVIS at the end of the pre- and post-criterion blocks. Next COVIS was simulated for an extended time (100 blocks), with the learning mechanisms disabled, using the saved pre- or post-criterion weights. The act of ‘freezing’ COVIS at the end of the pre- and post-criterion blocks enabled us to estimate ALCOVE’s long-run competence given the pre- or post-criterion state of the model.

**Fig 9:**
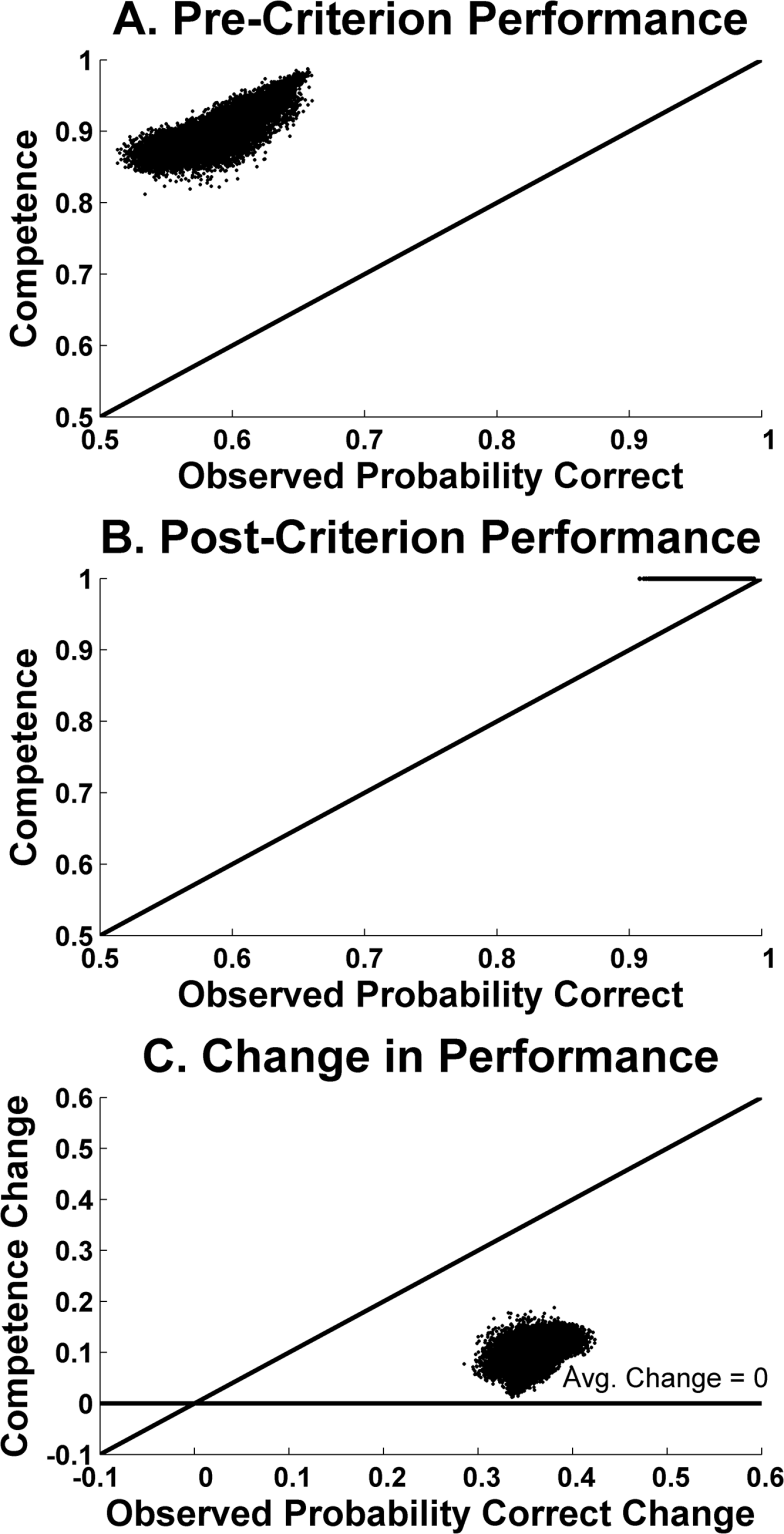
Results from the COVIS simulations, with COVIS learning the Fig 3 RB categorization task from a variety of parameter configurations. *A*. Underlying pre-criterion competence (Y-axis) plotted as a function of the pre-criterion performance actually expressed within the simulation (X-axis). *B*. Underlying post-criterion competence (Y-axis) plotted as a function of the pre-criterion performance actually expressed within the simulation (X-axis). *C*. The change in underlying performance competence (Y-axis) plotted as a function of the change in performance actually expressed within the simulation (X-axis). In all panels, the diagonal line represents perfect correspondence between the observed performance produced by the simulation and its underlying true competence at the point of producing that observed performance. Each data point represents the average data from one of 23,040 simulated competency transitions. All simulations were performed using Matlab and the code is available as supporting information.

The results were similar to the results from ALCOVE. Fig 9A (X-axis) shows that COVIS did find pre-criterion blocks of performance with low performance. This is caused by the truncation problem inherent in the definition of criterion. Confirming this cause, Fig 9A (Y-axis) shows that in reality COVIS has high true competence pre-criterion if the model is run fixed in its pre-criterion state. Fig 9B shows that COVIS’s post-criterion behavior is stable whether measured over one block or many. The definition of criterion in these simulations (and in Figs 4, 5, and 6) required that high performance be sustained, and so this convergence is predictable. COVIS cannot enter criterion unless its configuration is one that will produce long-run high performance levels. Fig 9C shows the crux of the issue—that is, the true transitions that COVIS engenders pre- to post-criterion. These transitions are small. COVIS, though it is a rule-learning model that envisions explicit hypothesis testing, also qualitatively fails to reproduce the dramatic learning transitions that humans show.

As with ALCOVE, COVIS’s gradualism is not an artifact of the parameter ranges tested in these simulations. Rather, gradualism arises intrinsically from the way that COVIS adjusts its weights quantitatively and gradually. In COVIS’s case, these mechanisms operate to adjust attention and to place the decision criterion optimally along the continuum of the chosen dimension. Like ALCOVE, COVIS might well fail in the way analyzed here across nearly the whole range of its parameter space. Indeed, many models that incorporate incremental learning algorithms—ALCOVE, COVIS, and others—will inevitably predict gradual changes in performance and fail to capture humans’ dramatic shifts in rule-based tasks. This limitation of standard gradient-descent algorithms (and likely incremental learning algorithms more generally) holds when examining trial-by-trial changes in the decision criterion [23].

Possibly, COVIS might succeed if given rule-based tasks that use binary-feature dimensions (i.e., criterial-attribute tasks). Then there would be less demand on learning the decision criterion, and gradient descent less of an issue. Indeed, early models of rule learning successfully demonstrated large shifts in performance with binary-valued dimensions [31]. But even so, COVIS cannot avoid serious criticism (and note again: this is self-criticism). For it is not a sufficient formal description of rule-based category learning if the model explains psychological transitions in one class of rule task while completely failing to do so in another. It is also not that clear that humans’ psychological transitions are that different between these tasks. In the end, what is required is a comprehensive psychological understanding of rule realization and discovery. At present, neither ALCOVE nor COVIS provides this understanding or even an appropriate formal description. Ideally, this understanding will be well motivated psychologically or well grounded in cognitive neuroscience, and it will attend not only to the why of sudden learning but also the when of sudden learning.

## Discussion

Forward learning curves remain common in empirical and modeling work on categorization. Indeed, in the debate about multiple systems and rule-based category learning, forward curves dominate the literature [10, 12, 32, 33]. In these four articles alone, *84 forward learning curves* were the focus of analysis, modeling and discussion. Regarding rule-based categorization, it is time for the field to move decisively beyond this analytic approach that averages away important psychological transitions, rendering them empirically invisible and theoretically avoidable, and instead fostering the appearance of gradualism in learning and the appearance of suitability of incremental learning models. In fact, the strong dominance of forward curves that has existed in the literature makes it clear how important it is that the theoretical conclusions of the present article be present in the literature as a counterweight. Humans’ psychological transitions in rule tasks must not be left invisible or avoided as we seek a psychological understanding of rule-based categorization. Those transitions may be the crucial observation in gaining that understanding.

BLCs are one analytic approach that can highlight humans’ psychological transitions in rule tasks. BLCs were the original contribution of Hays [21], though subsequently this technique and its importance have essentially been lost from the categorization literature. BLCs were perfectly suited to our goals here, and they are highly relevant to analyzing performance in many categorization studies, especially those involving hypothesis testing and rule learning. We contributed a new sophistication to the use of BLCs by describing the statistical-distributional problems that attend them and by providing the means for managing those distributional effects. These effects are minimal in the case of humans learning rule-based category tasks, because humans’ psychological transitions are so dramatic. But they were prominent in our modeling studies, so this issue requires modelers’ careful attention. We demonstrated the promising technique of freezing the model’s state to measure stably its long-run competence, thus eliminating distributional and sampling problems.

One need not adopt the BLC approach used here. There are other approaches for analyzing individual differences in category tasks [17–20, 23, 34–39]. A full review of these approaches is well beyond the scope of this paper, but all may provide compelling alternatives to forward learning curves in the investigation of rule-based categorization. The important thing, though, is that one’s approach must fully bring to light humans’ psychological transitions in rule-based tasks.

Analyzed appropriately, humans’ rule-based category learning shows sudden psychological transitions. As was recognized in early studies of rule learning [31], the obvious possibility is that humans suddenly discover the category rule that will work from among hypotheses consciously entertained with insights consciously realized. It is difficult for some to accept this possibility, but it is also difficult to provide another explanation of the BLCs in Figs 4 and 5.

This article thus raises several important issues for categorization science. First, everyone acknowledges that in many situations—classical conditioning, the learning of operant discriminations, paradigms fostering implicit-procedural learning, and so forth—gradual improvements in learning do occur. In categorization, some explain gradual learning trajectories using an exemplar-based system [15]. Others explain these trajectories using a striatal learning circuit by which responses are connected to coherent regions of representational space [40]. Our article is neutral on this point—crucially, it transcends these mechanisms by pointing to a second, profoundly different kind of learning. Our psychology of categorization must describe this form of sudden learning too.

Second, although data from cognitive and cognitive neuroscience studies of categorization suggest that humans have a dissociable utility for rule-based category learning [9, 41–45], our results make it plain that multiple-systems theorists are not correct as a formal-modeling matter. We saw that the rule-based component of COVIS qualitatively failed to reproduce humans’ dramatic learning transitions. Thus, rule theorists and modelers are still far from understanding the suddenness of rule discovery. These abrupt transitions are brought into sharp relief using backward learning curves. They leave rule theorists and rule modelers with a lot of explaining to do.

Third, exemplar models were subject to the same failure. Extensive simulations showed that the exemplar model ALCOVE also qualitatively failed to reproduce humans’ learning transitions. Exemplar models more broadly construed—that is, beyond their network versions—would also seem unlikely to be able to reproduce these transitions. All exemplar models conceptualize and analyze learning curves by assuming gradual changes in the breadth of exemplar experience (and exemplar storage), and by gradual increases in sensitivity (similarity acuity), gradual shifts in attention, and so forth. But the changes in Figs 4 and 5 have no gradualness. It seems unlikely that exemplar models would be able to explain why so sharp a change occurred at Block 0, or why there were minimal changes in sensitivity or attention until Block 0, or why sensitivity and attention suddenly surged at Block 0. Humans are probably not—as they learn in rule-based tasks—building up exemplar experience gradually, or increasing sensitivity quantitatively, or re-allocating attention slowly. Rather, learning explodes on the scene.

However, these conclusions do not exclude a role for exemplar processes in describing much of humans’ category learning [46, 47]. They rule out a universalist-unitary exemplar-based system, but exemplar processing could still be a part of humans’ overall categorization capacity. Our view is that this issue and debate has never necessarily been all or none—though some have tried to enforce this necessity—and it need not be so now. We believe that strong theoretical progress could lie in the collegial acknowledgment of different fundamental kinds of learning to be explored and described psychologically. In the end, the goal of everyone is the full psychological description of humans learning category tasks and applying category knowledge.

We opted to focus on ALCOVE and COVIS because these are two popular models of category learning that incorporate very different assumptions. Thus, the inability of these models to adequately explain rule learning provides an important insight that does not appear to be driven by theoretical differences. Nevertheless, the field of competing models is quite crowded and we cannot rule out the possibility another model may have been more successful. For example, although ALCOVE was unable to produce large changes in competence, it may be that more recent descendants of ALCOVE that incorporate more sophisticated attentional learning mechanisms [48, 49] would have had more success.

Finally, and perhaps most important, the present article makes the theoretical statement that the class of incremental learning algorithms typically incorporated in categorization models—no matter their underlying theoretical orientation—are generally lacking in their ability to explain what happens when humans discover rules, when they achieve insights, when they suddenly realize. The current simulations reveal a pressing need for theorists to build models that can allow for sudden, qualitative psychological transitions in the underlying state of the cognitive system. It will be an important and exciting next step to envision psychologically and formally the swirl of trial memory and feedback evaluation that occurs in working consciousness to foster the great leaps that minds take in rule-based tasks. And if we complete that psychological description, it will be a small but significant step forward for theory in categorization.

## Supporting Information

S1 Simulation Matlab code used to run the ALCOVE simulations. (ZIP)

S2 Simulation Matlab code used to run the COVIS simulations. (ZIP)
